# Evaluating the quality of the public transport service during the COVID-19 pandemic from the perception of two user groups

**DOI:** 10.1186/s12544-023-00578-1

**Published:** 2023-03-09

**Authors:** Karzan Ismael, Domokos Esztergár-Kiss, Szabolcs Duleba

**Affiliations:** 1 Department of Transport Technology and Economics (KUKG), Faculty of Transportation Engineering and Vehicle Engineering (KJK), Budapest University of Technology and Economics (BME), Műegyetem rkp. 3., Budapest, 1111 Hungary; 2 Department of City Planning Engineering, Technical College of Engineering, Sulaimani Polytechnic University, Sulaimani, 46001 Iraq; 3 Institute of Mathematics and Informatics, University of Nyíregyháza, Nyíregyháza, Hungary

**Keywords:** Public transport, Service quality, Travel satisfaction, IPMA, IPA, COVID-19

## Abstract

The current paper focuses on a comparative analysis of both public transport (PT) and private vehicle (PV) users’ perceptions on the quality of the service. To detect the key components of PT attributes a new hybrid methodology is applied, combining the importance-performance analysis and the importance-performance map analysis. The proposed hybrid approach is simpler and more integrated than the existing methods in the literature. The sample comprises an online panel and a total of 1028 questionnaires for PV and PT users surveyed during the pandemic period in Budapest. The results of the applied methods show that among the different groups, the service hour, the proximity, and the frequency attributes are important and performed well in the years of COVID-19. On the other hand, the temperature and the cleanliness factors are not significant predictors of the PV and PT users’ general satisfaction. The obtained results can be used by local governments and authorities, who seek to identify areas to enhance the service quality of PT during movement restrictions in a pandemic wave.

## Introduction

The quality of public transport (PT) services seems to be a common problem in the development of transport systems [1]. To achieve the goals of sustainable development, studying the main factors which affect the efficiency of the PT systems is a subject of interest [2], and further research is required to understand those factors that impact the demand for PT [3]. In addition, attracting private vehicle (PV) users would contribute to the advancement of sustainable transportation [4, 5]. Previous studies primarily focus on PT users [6–8] and potential users’ viewpoints [9, 10] to evaluate the quality of the PT services. In addition, it is interesting to explore the PV users’ perception and satisfaction [11–14] because satisfaction with the use of a specific travel mode affects future mode choice [15–17].

Several methods have been applied to measure the quality of the PT services, for instance, the Structural Equation Modeling (SEM) [6, 14, 18] and the Bayesian Network (BN) [7], and the Multi-Criteria Decision-Making (MCDM) method [19, 20]. These methods have some problems, and their improvement is necessary to generate more accurate results [21]. Thus, the importance-performance analysis (IPA) is frequently applied in the transportation planning and PT service quality assessment [22–25]. However, there is a lack of published results from empirical studies focusing on the evaluation of service quality considering both PT and PV users’ opinions to achieve an efficient policy regarding PT improvements. According to the European Commission [26], passenger transportation has been influenced by the COVID-19 outbreak in the European Union (EU) [27]. Recent studies widely consider the effects of the coronavirus on behavioral changes [28, 29]. However, few studies evaluate the quality of PT services during the Coronavirus [30], and there is a need for more sophisticated investigations [31, 32]. For these reasons, the goal of this paper is to examine the perceptions of different traveler groups. To do so this paper answers the following research questions: (1) Are there significant differences between PV and PT user’s rating of service quality? (2) How do the perceived service attributes affect each users’ overall satisfaction? (3) What are the main reasons behind the low frequency of using PT in regular travel for each group? (4) How should the improvement of service qualities be prioritized from the PV and PT users point of view? (5) What is the influence of COVID-19 in assessing the PT service quality attributes from the perspective of PV and PT users?

To overcome the issues discussed above, the current study investigates the following main points:

- Applying quantitative analysis supplemented by an open-ended questionnaire.
- Developing an IPA and IPMA approach to identify the priority areas of improvement of the PT service attributes.
- Conducting a sophisticated analysis with the distinction between regular PV and regular PT users’ perspectives during COVID-19 period and compared to previous studies [10].

A significant advantage of the current study is that the local government and policymakers can use its findings to develop future PT projects considering possible movement restrictions due to a pandemic. In addition, the combination between the IPA and IPMA methods is clearer and more integrated than the existing models in previous literature. The applied hybrid approach can be useful for authorities in determining priority service attributes and recommending managerial implications under unusual circumstances caused by pandemic restrictions, which is the main objective of this paper. The rest of this study is structured as follows. The literature review is the main topic of Sect. 2. Samples and survey design are explained in Sect. 3. Section 4 elaborates the methodological approach used to analyze PT and PV users’ satisfaction with PT service. Section 5 presents the main results. Section 6 discusses the long-term policy implications, the limitations of the research, and future work. Section 7 summarizes the conducted work.

## Review of studies on PT service quality during COVID-19

Sustainable cities depend on efficient PT operation [33], which can be achieved by accurately measuring the service quality and using the results to enhance PT [34]. For instance, the level of passengers’ satisfaction is one of the main factors that impact PT [35]. Moreover, improving PT service quality attributes are the factors that affect the travelers’ satisfaction, such as price [36], frequency [37], and punctuality [38], consequently increase the PT demand [39]. Another research states that waiting time, cleanliness, and comfort are the most significant factors influencing PT satisfaction, whereas the behavior of the driver and the journey time are less crucial [40]. Table 1 presents studies conducted on the assessment of PT service quality in different contexts [41].

**Table 1:** Studies measuring the quality of PT services and travelers’ satisfaction

Reference	Study context	Transport mode	Involvement (e.g., PV user, PT user, Both)	Method*
[21]	India	Metro	PT	BN, PLS-SEM
[42]	Brazil	All modes	Expert, PT	MCDM, IPA
[43]	Spain	Bus	PT	BWM, OLM
[44]	Indonesia	Bus	PT, PV	IPA
[45]	China	Bus	PT	OLM, OPM, IPA
[11]	Portugal	All modes	Public	Qualitative method
[4]	Spain, Italy, Germany, Portugal, UK	All modes	PV	OLM
[8]	China	Bus and Metro	PT	SEM
[46]	Spain, Portugal	All modes	PV	SEM
[17]	Lebanon	PT, PV	PT, PV	OLM
[47]	Taiwan	PT	PT	SEM
[48]	Italy	Bus, tramway	PT	Ordinal measure
[49]	Bangladesh	Bus	PT	DCM
[50]	UK	Rail	PT	OLM
[51]	Canada	All modes	PT	OLM
[52]	China	All modes	PT	PLS-SEM
[53]	Vietnam	Bus	PT	PLS-SEM
[54]	Ghana	Bus	PT	SEM
[10]	Spain	All modes	PT, PV	OLM
[55]	Hungary, Budapest	All modes	Passenger, non-passenger, and government	MCDM

*BWM best worst method. *OPM* ordered Probit model, *DCM* discrete choice model, *PLS-SEM* partial squares—structural equation model

Policymakers in European countries are focused on improving PT services, but more studies are needed to consider the quality of the PT services during the pandemic situation [31]. Mostly, the service quality of PT is examined by two main techniques: aggregate and disaggregate methods. Multi-criteria decision-making techniques are aggregate methods that aim to attain an overall index to show the assessment of the quality of PT and solve complex transport problems [19]. Conversely, the individual perception and the latent variables can be analyzed by disaggregate techniques, for instance, with discrete choice model [49, 51] and SEM [8, 21, 53, 54].

Another important point is the respondents’ involvement. In previous studies, most participants include PT users [35] and potential users of PT [56]. However, researchers and transport operators are also interested in investigating PV user’s opinion about the quality of the PT services. Consequently, more effort should be paid to examine the PV users’ needs, especially those who have experience of using PT [46]. This is considered as a research gap where the two groups of evaluators are not compared. Several different methods, including the IPA [44, 45], are applied for evaluating the quality of PT services, but only a limited number of papers perform importance-satisfaction analysis along with an ordered logit model (OLM) [43]. This is one of the first studies that integrates the IPA and the IPMA to detect the specific importance and performance attributes.

Previous studies measuring the quality of PT services usually consider the situation before the COVID-19 pandemic and assess the service aspects differently [57]. For example, safety is examined from two aspects: accidents and crime. Considering these factors, traveling by PT is safer than using PVs [58]. Based on mobility statistics and PT counts, there was an extraordinary interruption to the transport industry among other industries, during the pandemic [59]. It was found [60] that during COVID-19, the rail passengers’ perception changes and shows a lower score of comfort level in crowding while traveling. Besides, to reduce the spread of the virus, the authority of South Korea warned against using the PT and encouraged other forms of traveling [61], thus is foreseen that the use of PT may decrease significantly in longer term [62].

Recent research findings show that during COVID-19 people chose not to travel, they were more likely to stay at home and less likely to visit the city center. As a result, there has been a reduction in crowding in congested areas of up to 90% [63]. Moreover, approximately 80% of global citizens decreased their outdoor activities, and the number of trips decreased by 68% due to COVID-19 [64]. A study concerning the spread of the virus in Colombia, reviews the short-term effects of various policies on the transport system [65]. Other researchers find that communication and the enforcement of safety measures are vital during the pandemic period [27]. A review article, considering the measurements of the COVID-19 and its impacts, examines 254 academic papers and finds that over 57% study the social impacts, around 17% deal with the economic and environmental impacts, and merely some papers analyze the measurement of passenger transport [32]. Several studies investigating how travel behavior changes as a result of the COVID-19 pandemic, such as the alterations in urban mobility [66] and mobility behavior [67] additionally, the mode choice [68].

Without exception, there was a significant reduction in using PT during the pandemic internationally and a negative perception regarding PT was experienced [29]. For instance, in Spain, there was 85% reduction compared with pre-COVID-19, and in Sweden, a reduction from 60 to 40%. Moreover, in Australia, PT trips decreased from approximately 15% to 7%, while in Germany by 70%, and in Budapest in early 2020 PT was reduced by 80% [69]. Another study considered the influence of COVID-19 on mobility patterns, including the PT system in Budapest, finding that PT users shift their mode by 31.5, 10.6, and 6.9 times more likely than those who drive a car, ride a motorbike, or walk to work. This result confirms that PT usage was perceived riskier than active modes and personal car usage [70]. Many nations, including Hungary, have suggested implementing restrictions on various levels and introduced social segregation measures [71]. For example, in Budapest it is mentioned that the lockdown initiated a substantial reduction in the number of PT passengers and interurban bus ticket and pass sales in 2020, along with the decline in commuter traffic in the city [72]. At the same time, the usage of private cars, cycling, and walking was significantly raised during COVID-19 in urban areas, while keeping the price of the monthly ticket [59, 72]. Apart from that, the level of service of the bicycle system remained the same as it was in 2019. It is also true that the capacity and frequency of PT was unaffected by COVID-19, while there was a significant decline in the number of travelers [73]. This paper represents the first study conducted in assessing the quality of the PT services while considering the impact of the COVID-19 pandemic from various viewpoints in Budapest.

## Survey design and sample description

The analysis's techniques are based on information obtained from a widely randomly disseminated survey among the citizens of Budapest through online and printed questionnaires. This study considers Budapest as a case study that is the capital of Hungary. We assessed the details of mobility options, including PV (i.e., e-scooter, bicycle) and PT (i.e., tram, metro, train, bus) to show an overview of the mobility system in Budapest. Over the past few years, urban mobility has altered as new motorized transport modes (such as e-scooters or e-bikes) have been integrated into urban transportation, and inventive ways to use the already existing ones have emerged (e.g., car-sharing, bike-sharing). At the same time, cities are investing heavily in PT to improve its appeal as well as particularly for users of private vehicles (PVs) [74] to cover the problems of using micro-mobility services (e.g., weather conditions, last mile). Additionally, encouraging the use of PT for those who rely on private vehicles is still crucial for attaining sustainable mobility in metropolitan settings [10]. To discover the context of Budapest, we provide some general characteristics of the city. Budapest's population is 1.75 million (53% female and 47% male) [73], with a population density of 3,366/km^2^ and a city area of 525.2 km^2^ [75]. The age range of the residents between 15 and 64 is 66%. In 2020, the average yearly net income per capita was approximately €6,400, whereas the national average was around €5,000.

According to the European Platform on Mobility Management (EPOMM), only four European cities with a population of over one million had a higher percentage of PT modal shares than Budapest with 45% [77]. At the same time, the motorization rate (passenger cars per 1000 inhabitants) is low. Eurostat shows that it was 516 in the European Union (EU) in 2018, whereas it was only 376 in Budapest. Only Vienna (with 374) and Berlin (with 330) had lower ratings among the main EU cities. These numbers demonstrate the importance of PT to the city [59]. In addition, Budapest provides an interesting case in Eastern Europe because it has 328 bus lines, 4 metro lines, 34 tram lines, 16 trolleybus lines, and 16 railway lines with 2073 PT vehicles in use per day, which provides a vivid and dense transport network [78]. The Hungarian Central Statistical Office revealed that in 2019 the number of passengers carried by bus, tram, trolleybus, metro, and rail was 653.3, 417.2, 84.7, 354.0, 60.6 million, respectively. However, on average these numbers significantly decreased from 1,569.8 in 2019 to 1,021.6 in 2020 [79].

The bike-sharing system in Budapest opened in 2014, and in 2020 it had 160 stations and around 2,000 bicycles [73]. In terms of e-scooters, the service was launched in Budapest in the spring of 2019. The infrastructure in Budapest is adequate for e-scooters with an extensive network of bike lanes and trails. While the target audience includes not only the inhabitants but also many tourists. Currently the number of scooters is around 300 vehicles [75]. The length of cycling infrastructure in Budapest (325 km) is higher than in other Europe capital cities (i.e., Rome, Lisbon, and Vilnius) that is also beneficial for e-scooter usage. It can be stated that the number of users increased during the pandemic [75]. Moreover, in Budapest, ride-hailing (Bolt) was established in 2016. The years between 2016 and 2020 were marked by a thriving tourist sector, particularly Bolt drivers and customers benefited significantly from this trend. Nevertheless, ride-hailing initiated the shift from a taxi service to a web-based food courier during the COVID-19 pandemic [76].

In order to capture the perceptions and travelers’ satisfaction, the survey was adopted between October and November 2020 during COVID-19, and a total of 1028 answers were registered. The collected data is separated into classes: 500 PV users, who use motorized vehicles (i.e., car, motorcycle, or scooter) for their daily journeys as well as use PT at least occasionally, and 528 PT users. 81 participants fail to complete the survey correctly, and consequently, they are excluded from the data set resulting in a total of 947 adequate respondents. Specifically, 455 respondents (48%) define themselves as regular PV users, and 492 respondents (52%) are regular PT users. The questionnaire is designed to include the following parts: socio-demographic characteristics, mobility characteristics, and questions regarding the participants’ satisfaction. Additionally, the survey includes questions contributing to the identification of the target population, about PV usage and the users’ experience and satisfaction with PVs, and regarding the main reasons for using PT less frequently. In literature there is no consensus regarding which service attributes should be considered for assessing PT [80] because service quality is a complex and abstract concept [36]. For example, past research used almost identical service quality parameters as those in the study by Tyrinopoulos and Antoniou [39]. It is worth mentioning that a review article argued that the most well-known attributes could be assigned as follows: physical attributes (including reliability, frequency, accessibility, price, speed, information, provision, vehicle condition, and easy transfer) and perceived attributes (including safety, comfort, convenience and aesthetics) [36]. Similarly, another review study on PT service quality suggested using the following attributes serving the goal of assessing the quality of any PT service, such as affordability, times, safety, accessibility, reliability, fares, intermodality, information, ticket price, frequency, space inside the vehicle, cleanliness of the vehicle and accessibility, employee service, availability of facilities, reservation, security and safety in terms of the record of accidents [81]. It has been noted that most of the service attributes used to assess PT modes are similar. Therefore, based on an extensive literature review, expert opinions, and the researchers’ judgement, 14 quantitative urban PT quality attributes were chosen for the survey after some alterations in the design to reflect local realities, unique features of local transportation network, and the goals of the transport operators [4, 10, 14, 36, 39, 46, 80, 81].

**Fig. 1:**
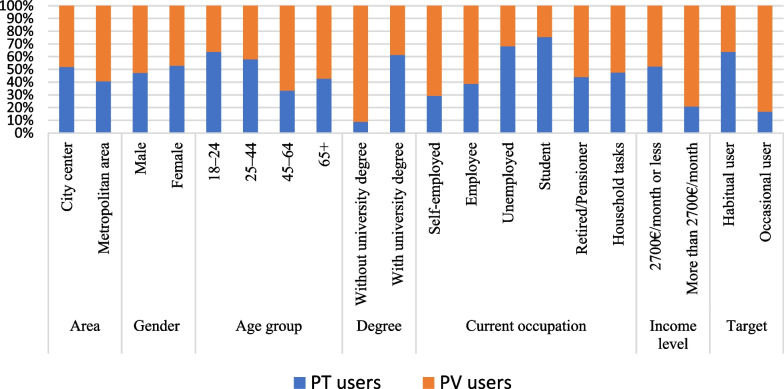
The socio-demographic characteristics of the PV and PT users

A preliminary test on the 947 respondents is applied to demonstrate the adequate reliability and validity (Cronbach’s α = 0.901) of the questionnaire. The one-sample Kolmogorov–Smirnov (K-S) is implemented to test the normality of the perception by using IBM SPSS 25 [82]. The results demonstrate that the absolute value of K-S ranges from 0.169 to 0.274 with *p*-values as 0.000 (< 0.05) showing the data to have a non-normal distribution. Fig. 4 in the appendix provides supplementary evidence for the non-normal data. Figure 1 presents the socio-demographic characteristics for both types of users. Overall, the distribution of the age groups is varied for the user groups. In the case of the age group 18–24, 60% of the respondents use PT, while 35% are PV users. The distribution of the age group 24–44 years is almost the same for both types of users. The percentage of the users aged 45–64 years and 65 + years is higher for the PV users than for the PT users in those age groups (i.e., approximately 70% and 60%, respectively). It can be seen that the proportions of the male and female groups are the same, at around 50%, for both types of users. Interestingly, respondents living in the city center are evenly split (50% each) between using PV and PT, whereas in the metropolitan area, the proportion of the PV users (60%) is higher compared with the PT users (40%).

Predictably, less than 10% of the respondents without a university degree use PT with more than 90% of this group using PVs. However, among university degree holders, 62% use PT, and solely 38% travel by PVs. Regarding the frequent mobility usage in a normal week, more than 60% of the habitual users are PT users. However, in terms of occasional usage, the percentage of the PV users is higher (85%) than that of the PT users. This reflects that the PT users include more students and unemployed respondents, while PVs are more likely to be used by those who are employed or self-employed. Generally, people with low monthly household income prefer PT, and a high percentage of respondents with high income (i.e., more than 2700 €/ month) use PVs. The results are in line with the general census data, where an almost equal proportion of gender for all ages can be discovered based on the distribution of Hungarian population in January 2020, except from the age group above 65 years old people, where the percentage of the women are greater than men. In addition, Hungary has the majority of inhabitants among (24–45) year old people [79].

In a satisfaction survey, the PT and PV users are asked to rate their satisfaction with the PT service in relation to 14 variables using a conventional psychometric measure (i.e., a 5-point Likert scale ranging from very dissatisfied (1) to very satisfied (5)) [83]. On the same scale, the participants are also asked about their overall satisfaction. Table 2 depicts the two types of users’ mean and standard deviation regarding the perception of the service quality attributes and one general satisfaction item obtained from a recent study [10].

**Table 2:** The descriptive statistics of the indicators by the two types of users

Code	Variable	PT user		PV user	
		Mean	StdDev	Mean	StdDev
S1	Service hour	4.02	0.92	3.64	1.03
S2	Proximity	3.98	0.84	3.64	0.98
S3	Frequency	4.05	0.91	3.71	0.99
S4	Punctuality	3.81	0.98	3.46	1.00
S5	Speed	3.73	0.95	3.32	0.99
S6	Cost	3.80	1.14	3.19	1.15
S7	Accessibility	3.81	0.99	3.26	1.15
S8	Intermodality	3.80	0.96	3.42	1.07
S9	Individual space	3.19	1.10	2.80	1.08
S10	Temperature	3.06	1.12	2.52	1.05
S11	Cleanliness	2.88	1.15	2.46	1.06
S12	Safety	3.76	0.99	3.57	0.97
S13	Security	3.36	1.03	3.10	1.02
S14	Information	3.67	1.03	3.57	0.96
GS	General satisfaction	4.14	0.89	3.65	1.05

## Methods

In the first step, the focus is on the preparation of the database, so that the data are appropriate for further analysis. The IBM SPSS statistics version 25.0 is used to perform the data analysis [82]. To determine service quality factors, exploratory factor analysis (EFA) is applied by using principal component analysis (PCA) with Varimax rotation at an eigenvalue > 1 on the 14 perceived service quality indicators. In accordance with the literature, those indicators with a factor loading ≥ 0.5 are extracted [13]. This technique can be a useful selection procedure for further analysis by decreasing the dimensionality of such datasets.

It is highlighted that identifying the normality of data is vital before doing the primary analysis. Based on this outcome, we can use either a parametric test (i.e., one-way ANOVA, T-test) if the data is normally distributed, or non-parametric tests in case of a non-normal distribution. Non-parametric tests include the Wilcoxon signed rank test, Mann–Whitney U, and Kruskal–Wallis. In this study, a nonparametric test, the Mann–Whitney U test is applied because the factor scores are not normally distributed (Figure 4). A nonparametric (e.g., Mann–Whitney/Wilcoxon) test compares the distribution across two independent groups, with the dependent variable being ordinal and non-normally distributed [84]. Afterward, based on the selected attributes, the OLM is applied for examining the association between the 14 items as independent variables and 1 item as dependent variable [85]. The OLM is appropriate for analyzing the variables with a ranking order [47]. An ordinal scale is used to present the respondents’ overall satisfaction level with the PT service. Ordinal scales have several distinct characteristics: the order level is clear, there is an unknown absolute distance between the levels, or there is a potential for unequal distances between the adjacent ratings.

- In the OLM, Y is an observed variable which is a function of unmeasured variable (Y*).
- The unobserved variable Y* has different threshold points as shown by the formulas [86].
  1 $${\text{Yi }} = { 1}\quad if\;Y^{*} i\,{ }is\,{ } \le {\text{ k}}_{{1}}$$
  2 $${\text{Yi }} = { 2}\quad if\;{\text{ k}}_{{1}} < Y^{*} i \le {\text{ k}}_{{2}}$$
  3 $${\text{ Yi }} = { 3} \quad {\text{if}}\;{\text{k}}_{{2}} < Y^{*} i \le {\text{ k}}_{{3}}$$
  4 $${\text{Yi }} = { 4} \quad {\text{if}}\;{\text{ k}}_{{3}} < Y^{*} i \le {\text{ k}}_{{4}}$$
  5 $${\text{Yi }} = { 5} \quad Y^{*} i {\text{ > k}}_{{4}}$$

Hence, Y* can be formulated as follows:

6 $$Y_{i}^{* } = \mathop \sum \limits_{k = 1}^{k} \beta_{k} X_{ki } + \varepsilon_{i} = Z_{i } + \varepsilon_{i}^{{}}$$

The error term represents the possibility of imperfect measurement of the variables and the potential absence of some important variables from the equation. The Maximum Likelihood approach is used to estimate the β parameters, and Nagelkerke R2 is used to assess the OLM's degree of goodness-of-fit. [87]. Based on the Wald test *p*-values, the statistical significance of the variables is determined.

Finally, the IPA and the IPMA are applied to identify the priority of the significance attributes. It is worth mentioning that to support the results of the quantitative analysis, a qualitative analysis is conducted. Previous studies apply the IPA to find the relation between importance and satisfaction, for example, to evaluate the quality of the PT services and passenger satisfaction [85, 88], and to perform importance-satisfaction for evaluating the quality of transit services [50], while other studies apply the IPA and the IPMA to assess the quality of the PT services [22, 89]. Based on the literature, the IPA is broadly used as a tool in practice, for instance in public administration, recreation, and tourism [23]. The IPA technique is applied for the assessment of service quality; usually, it is used in questionnaires related to satisfaction [49].

Many types of research have examined the satisfaction of ridership to achieve important service attributes and/or specify development priorities. The IPA is able to provide both targets. Echaniz et al. [50] state that the results of conventional techniques identifying important service attributes might be biased which is limited by this problem. A study explores that ordered logit model is assumed to be a more reliable instrument for decision making compared to the traditional ratings [90]. For IPA, the current paper uses ordered logit models to gain the importance of 14 attributes, because overall satisfaction is evaluated on an ordinal scale. The means of the attributes and the coefficient of significant values are calculated to draw the IPA.

The IPMA is referred to as a priority map analysis or an importance-performance matrix analysis. It is a powerful technique to show the results in a graphical way [21]. The IPMA usually ordered the greater interest of target construct, satisfaction is an example. In the satisfaction surveys, the obtained results are used to make two-dimensional maps, where the horizontal axis (i.e., x-axis) represents the performance and the vertical axis (i.e., y-axis) represents the importance. The IPMA can support the analysis. More precisely, the IPMA does not only analyze the path coefficients (i.e., the importance dimension), but also considers the average value of the latent variables and their indicators (i.e., performance dimension) [91]. There are several steps to be considered when applying an IPMA for an analysis. In the first step the requirements should be checked (i.e., rescaling and coding the latent variables, which must have the same scale, and the outer weight estimates must be positive). After fulfilling the requirements, step 2 proceeds with the analysis of latent variables and the computation performance values, while in step 3 their importance values are calculated. In the last step the importance-performance map is created [91]. Some researchers consider open-ended questionnaires as qualitative data, where respondents can openly show their expression or, more commonly, dissatisfying with the service [92, 93]. A review article, on the other hand, examined qualitative studies and omitted open-ended questions as a qualitative method that asked as a supplement to a large-scale, quantitative study [94].

The survey design in transportation planning is crucial for collecting sufficient data. Transport planners and agencies commonly use data to assess passenger perception and satisfaction. However, the survey design process is complicated, time-wasting, expensive, and needs caution to reduce biases. Specifically, the ordering of questions or wording can thoroughly influence the respondent's answer [95]. Most survey questions are closed-ended with limited possibilities for respondents to pick, but in this case the data are simple to gather, code, and analyze. Hence these are thought to be effective. In the survey technique, where researchers seek to understand the opinions or experiences of a representative sample to generalize to a larger population, efficiency is crucial. Collecting data from huge numbers may be necessary to assure the accuracy of estimations [96]. Nevertheless, by failing to collect information about unexpected issues, poorly designed or closed-ended surveys may lead policymakers to ignore crucial aspects of real or perceived service quality [97].

To solve this, scholars have suggested user-oriented qualitative methods (i.e., including open-ended questions to allow respondents to express their perception freely) to gauge perceived service quality [93]. By doing this, the PT system will be able to target service-related issues more efficiently and will be able to present a complete overview of its performance. Consequently, the decision-makers can benefit from the results of these surveys enabling them to create better policies that are beneficial for specific segments of people as well as valuable for the whole populations [98]. Consequently, in the present study, PV and PT users were asked to mark the three main reasons out of twelve questions that explain the low user frequency of public transport in their situation.

## Results

### The results of the PCA and the descriptive statistics

The first step of the analysis procedure is applying the descriptive analysis and PCA. As shown in Table 2, the passengers of PT have a more positive opinion on all indicators compared with PV users. The most appreciated aspects of service quality according to PT users are service hour, frequency, and proximity. Similarly, in the case of the PV users, frequency, service hour, and proximity scores are high. In general, both types of users are content with service quality: PT users with a score of 4.14 and PV users with 3.65. Interestingly, the cleanliness has lower scores (i.e., 2.88 and 2.46) for both the PT and PV users. Table 2 The descriptive statistics of the indicators by the two types of users.

Table 3 presents the results of the PCA (loading each variable by the two types of users). The loadings lower than 0.5 are excluded from the analysis; thus, all loadings are greater or equal to 0.5. The variables for the PV users are divided into three factors (i.e., components); however, the variables for the PT users are minimized to two factors. In the analysis, those values are chosen that have higher loading factors.

**Table 3:** The PCA for measuring the perceived service quality and travelers’ satisfaction

Code	Component 1		Component 2		Component 3		*p*-value
	PV user	PT user	PV user	PT user	PV user	PT user	
S1		0.804	0.699				0.000
S2		0.765	0.807				0.000
S3		0.791	0.797				0.000
S4	0.510	0.736					0.000
S5	0.563	0.700					0.000
S6	0.569	0.555					0.000
S7	0.626	0.500					0.000
S8	0.594			0.501			0.000
S9	0.712			0.727			0.000
S10				0.779	0.550		0.000
S11				0.823	0.659		0.000
S12				0.569	0.759		0.002
S13				0.652	0.694		0.000
S14				0.522	0.586		0.041

Based on the results of the PV users, the correlation among the variables is not higher than 0.8, and there is no negative relationship. This endorses that the data do not have multicollinearity with a determinant of 0.005 > 0.00001. This is the first evidence suggesting that the PCA can be run. Additionally, KMO = 0.880, being > 0.5, which confirms that the adequacy of the sampling is acceptable. Furthermore, Bartlett’s Test reveals that the correlations between the items are large enough for the PCA with significant sphericity (X2 (91) = 2334.456 and *p*-value = 0.00, being < 0.05). Another important confirmation is the commonalities in which all extractions are greater than 0.3, as suggested in the literature [99]. Regarding the PT users, the sampling adequacy is acceptable (KMO = 0.910), and Bartlett’s Test of sphericity confirms that the correlations between items are large enough for PCA (X2 (91) = 3073.881, *p*-value < 0.001, and determinant = 0.002). Table 3 also displays the results of the Mann–Whitney/Wilcoxon test for all variables. An attribute with a *p*-value < 0.05 shows that there is a significant difference between the perception of the PV and PT users. The *p*-values demonstrate that all indicators are significant; thus, the differences in the perception of the types of users regarding the service quality is proven.

Figure 4 in the appendix illustrates that the PT users have a better perception of almost all the indicators once compared with the PV users.

### The results of the OLM

The data is analyzed by using statistical software StataMP 16. Before running the OLM, the variance inflation factor (VIF) is calculated. In a regression model, when two or more independent variables are highly correlated, it results in multicollinearity. If multicollinearity occurs, it might cause a problem, because it would be not possible to recognize the individual influences of the independent variables on the dependent variable. Table 4 shows that the VIF is less than 5 (i.e., the cutoff) for each independent variable demonstrating that there is no multicollinearity between the independent variables [100].

**Table 4:** The collinearity results of the independent variables

Code	VIF	
	PV user	PT user
S1	1.77	2.20
S2	2.19	1.96
S3	1.88	2.21
S4	1.81	2.29
S5	1.68	2.21
S6	1.37	1.51
S7	1.68	1.75
S8	1.91	1.90
S9	1.70	1.82
S10	2.04	1.93
S11	1.90	2.21
S12	1.54	1.86
S13	1.82	1.77
S14	1.36	1.44

Table 5 presents the results of the OLM**.** Four service indicators (i.e., service hour, proximity, cost, and accessibility) are significant at 5% confidence level for PV users, and three indicators (i.e., intermodality frequency and information at 5% confidence level) are significant for PT users. Furthermore, the results show that service hour has the largest coefficient (0.744) for the PV users, while frequency is the largest in the case of the PT users (0.484). In the model fit information, according to the Pseudo R^2^ indicator, service quality attributes explain 17.40% and 15.09% in overall satisfaction variability in the PT and the PV models, respectively.

**Table 5:** The results of the OLM for the respondents’ satisfaction of PT and PV users

	Coefficient		*p*-value	
Code	PV user	PT user	PV user	PT user
S1	0.744	0.194	0.000^**^	0.180
S2	0.459	0.088	0.001^**^	0.563
S3	0.002	0.484	0.988	0.001^**^
S4	0.105	0.134	0.382	0.331
S5	− 0.180	− 0.094	0.133	0.502
S6	0.422	0.174	0.000^**^	0.072
S7	0.317	0.082	0.002^**^	0.495
S8	− 0.042	0.275	0.714	0.034^**^
S9	0.006	0.059	0.950	0.595
S10	− 0.088	− 0.002	0.483	0.986
S11	− 0.006	0.179	0.960	0.122
S12	0.094	0.217	0.412	0.076
S13	0.154	− 0.092	0.198	0.428
S14	0.084	0.281	0.455	0.007^**^
Nº obs (N)	455	492		
LogLikelihood with zero coefficients	− 630.61869	− 580.1657		
Final LogLikelihood	− 520.90402	− 492.60259		
Pseudo R2	0.1740	0.1509		

^**^ Significance at 5%

### The open-ended questionnaire analysis

To identify the differences between the PV and PT users’ opinions, an open-ended questionnaire as part of the survey analysis is performed, which focuses on explaining the main reasons behind a low frequency of using PT in regular travels. Table 6 shows the PV users, approximately half of the people (48%) need a car to transport the kids to school. In contrast, solely 15% of the PT users think that they need a car for this purpose. For both users (i.e., PV and PT), the second-highest percentage (41% and 38%, respectively) reason is ‘"It's unclean, uncomfortable, noisy, and the temperature isn't suitable,"’. Predictably, more than one-third of the PV users (38%) prefer to use the car, while in the case of the PT users, this value is solely 13%. 35% of the PV users vote for ‘PT takes a long time to get the destination’ compared to one-third (30%) of the PT users. Regarding ‘There is no adequate service for my route, a similar proportion of the PV and PT users (25% and 23%, respectively) select this reason. Moreover, the reason ‘Stops are far from my starting point or destination’ is chosen by a higher percentage of PT users (23%) than PV users (17%). From both groups of users, solely a small proportion of people choose the reasons ‘I do not know the service’, ‘There is no PT’, ‘It’s unsafe’ or ‘Expensive’.

**Table 6:** A comparison between the PV and PT users explaining a low user frequency of PT

		Frequency PV user	%	Frequency PT user	%
1	I need the car to take the children to school	220	48	72	15
2	It's unclean, uncomfortable, noisy, and the temperature isn't suitable	186	41	189	38
3	I prefer to use the car	174	38	66	13
4	It takes a long time to get the destination	158	35	148	30
5	There is no adequate service for my route	112	25	111	23
6	I don’t like PT	90	20	39	08
7	The stops are far from my starting point or destination	78	17	112	23
8	The transfers don’t work well	59	13	69	14
9	It’s expensive	43	09	34	07
10	It’s unsafe	33	07	39	08
11	There is no PT	14	03	31	06
12	I don’t know the service	08	02	24	05

### The results of the IPMA

**Fig. 2:**
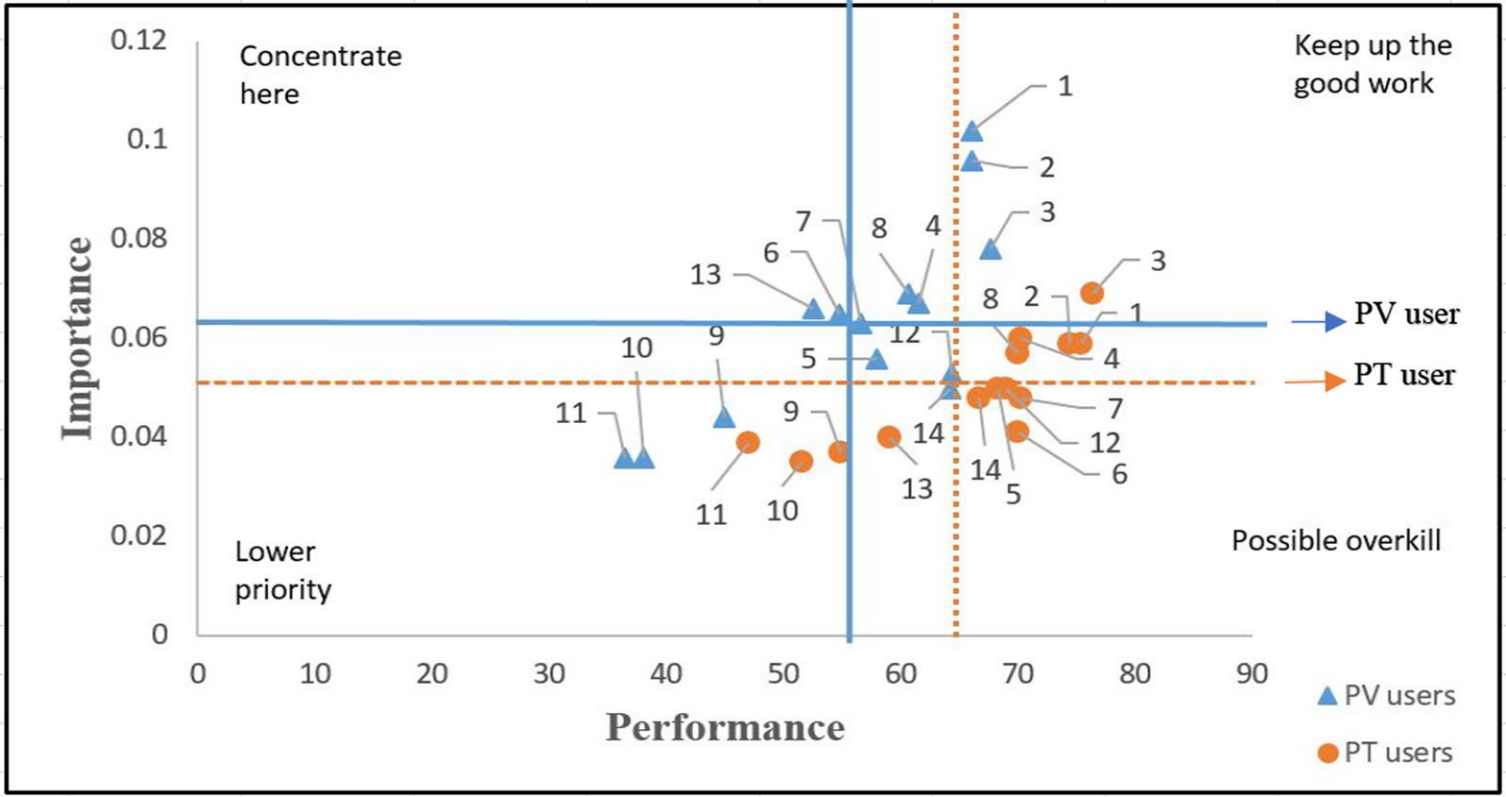
The two types of transport users’ IPMA results for the PV users (blue) and the PT users (orange) colored in the vertical and horizontal lines

This study performs an IPMA by using Smart PLS 3 between the two different user types for significant values. All variables applied to the IPMA are obtained from the results of the PCA shown in Table 3. Figure 2 presents the results of PV and PT users’ IPMA that the importance index and performance index are presented separately colored for the PV users (blue) and the PT users (orange) in the vertical and horizontal lines, respectively. Consequently, the space is divided into four quadrants: Concentrate here is written in the first quadrant, "keep up the good job" is marked in the second, "low priority" is described in the third, and "potential overkill" is defined in the fourth [23, 90]. In the current research, the target construct defines the passengers’ satisfaction, which is predicted by 14 indicators. According to the literature, the main aim of the IPMA is to detect those indicators that present moderately low performance but high importance for the target constructs [101].

Looking at the results, there are two variables (cost and security) in the ‘concentrate here’ quadrant for the PV users. Service hours, frequency, and proximity are important, also punctuality and intermodality are relatively important having a higher performance for the PV users, which highlights the benefits of the provided service quality for the users. Results show that speed, accessibility, safety, and information perform well in the ‘possible overkill’ quadrant. Besides, individual space, temperature, and cleanliness, are in the ‘lower priority’ quadrant. This is potentially the case because of the lower ridership during the COVID-19 pandemic. To support these findings, see the results of the open-ended questionnaire analysis (Table 6) in Sect. 5.3.

According to the results, the PT users have service hour, proximity, frequency, punctuality, and intermodality classified in the ‘keep up the good work’ quadrant. This indicates that these factors provide great performance, and most of the passengers perceive them as important. Therefore, service availability aspects give an advantage to the PT service and are required to maintain stable over time. Furthermore, accessibility, speed, cost, safety, and information are grouped in the ‘possible overkill’ quadrant specifying that these factors are well-performed, and PT administrators need to retain adequate performance levels of these factors. Similarly, the PT users’ individual space, temperature, cleanliness, and security attributes are grouped into the ‘lower priority’ quadrant proposing relatively low importance and performance of these attributes. Thus, these factors require second-level priority actions by the PT operator.

### The results of the IPA

**Fig. 3:**
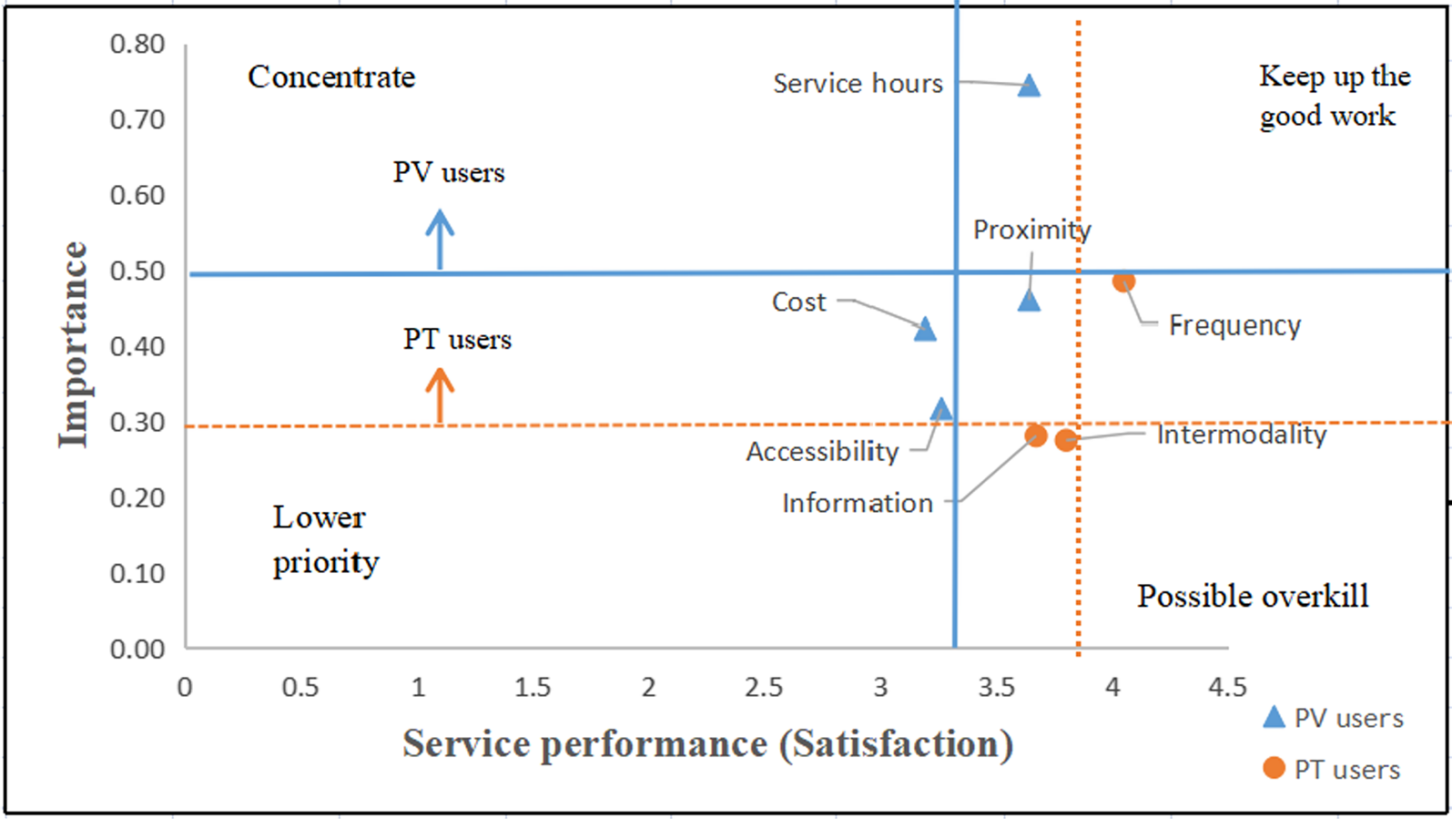
Importance—satisfaction/performance results for the PV users (blue) and the PT users (orange) colored in the vertical and horizontal lines

**Fig. 4:**
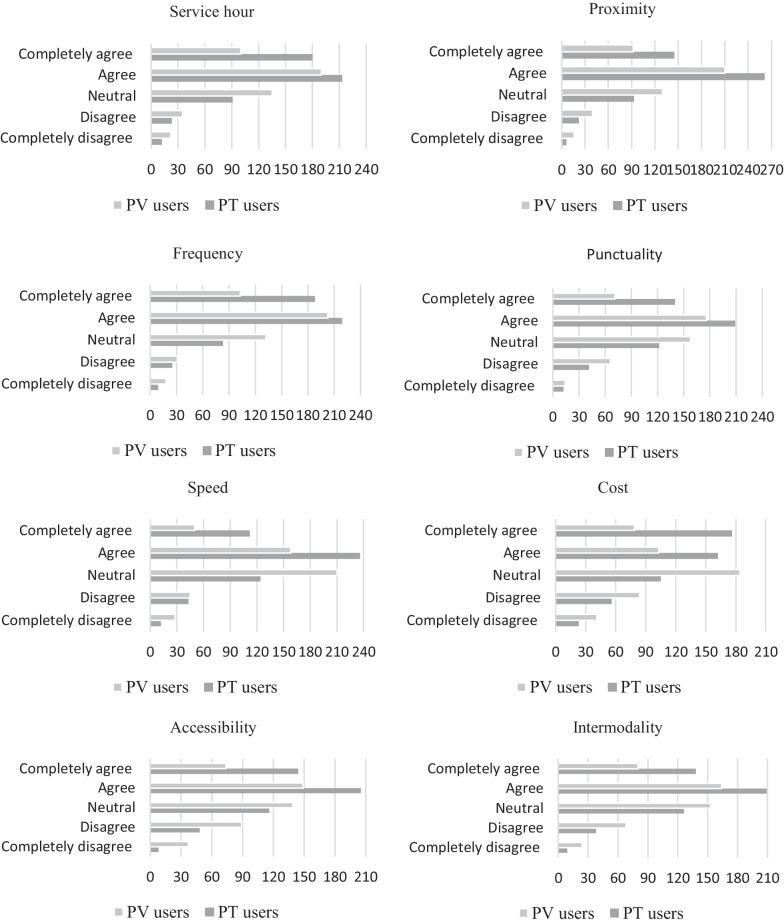

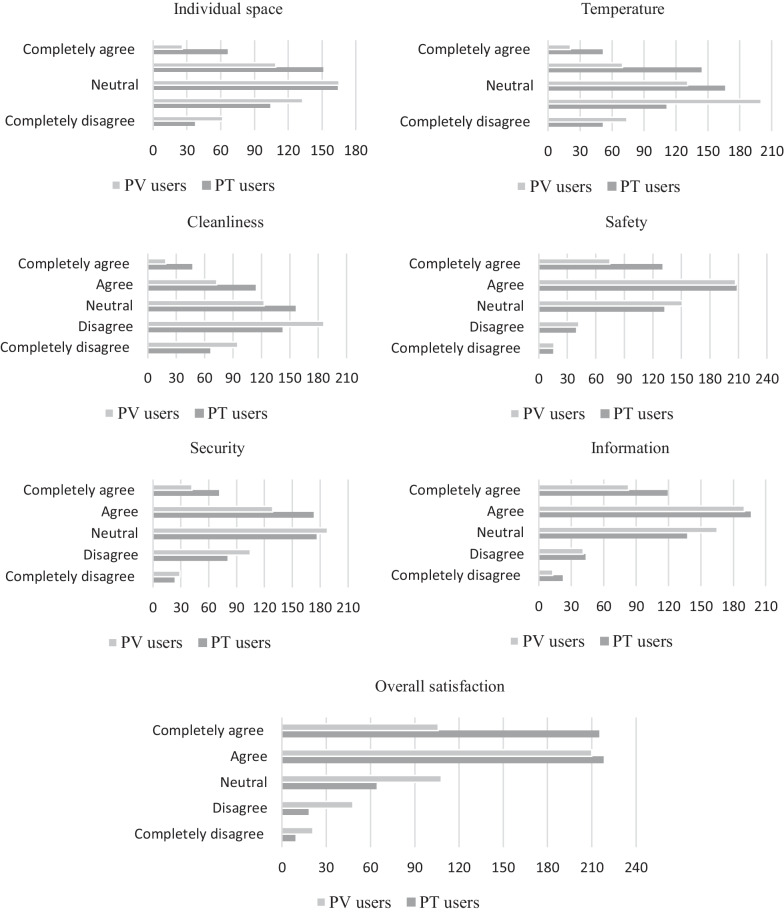
Frequency score distributions of th03030e PT and PV users’ responses

In this research, which is the first study of its type in this field, the OLM using the IPA and the IPMA with SmartPLS 3 are proposed to recommend specific policy measures. Following the application of the OLM for disaggregating the analysis (see the results in Table 5) and the mean rate for satisfaction in Table 2, the results show the importance-satisfaction quadrants for both PV and PT users. The importance index and performance index are presented separately colored for the PV users (blue) and the PT users (orange) in the vertical and horizontal lines. As shown in Fig. 3, only the significant attributes are represented. In the ‘keep up the good work’ quadrant is the service hour for the PV users and the frequency for the PT users, which are important and perform well. In the case of both PV and PT users, there are no attributes presented in the ‘concentrate here’ quadrant. Interestingly, the attributes in the ‘lower priority’ quadrant for the PV users are cost and accessibility, but for the PT users, these are intermodality, and information, which are unimportant, but moderately perform well. Finally, proximity is in the ‘possible overkill’ quadrant, which has relatively high importance and performs well. It is worth mentioning that overall, the performance levels of the PT user attributes are higher than those of the PV user attributes. This suggests that there might be more resources for proximity, intermodality, cost and information than needed.

## Discussion

In the current study, although several attributes need to be improved by service providers, almost half of the total attributes were found to have good performance when evaluated. It indicated that integrating IPMA and IPA techniques in a systematic manner was a potential approach to achieve robust results in measuring the quality of PT services. The obtained results can be useful for the local government of Budapest and the policymakers in enhancing PT projects. Although the present research is considered as a crucial step towards a deeper understanding of the service quality priorities to improve the PT system in urban areas, cautions need to be taken into account when generalizing its findings because of the unusual circumstances of the time of the survey. All conclusions on PT service developments are relevant in reflection of a pandemic situation (or for its precaution) but might be less relevant for a usual period. Nonetheless, the possibility of another pandemic period in the future has to be taken into account, and in this case, the results could provide some useful insights into the quality of PT service related to different user groups.

Based on the survey results, question (1) could be answered as an attribute with a p-value smaller than 0.05 in the Mann-Whitney/Wilcoxon test showing a significant difference between the perception of the PV and PT users. The results demonstrate that all indicators were significant. In accordance with earlier research, the results of the current study showed that PT users had a more positive perception than PV users [10, 11]. In terms of question (2) it could be acknowledged that all 14 service quality attributes had distinct relationships within overall satisfaction for both groups (i.e., the perception of the PT users’ satisfaction was higher than the perception of the PV users’ satisfaction regarding the quality of the PT services). Explicitly illustrated from the results of the OLM, four service indicators (i.e., service hour, proximity, cost, and accessibility) were significant and contributed to the overall satisfaction for PV users, while three indicators (i.e., intermodality, frequency, and information) contributed to the overall satisfaction for PT users. In the case of Budapest, it was found that people, in particular young (aged between 20 to 30 years old) in urban areas were less interested in using the private car [102]. This result confirmed that the PT system in Budapest is well-organized with multi-modal transport options and stops within an acceptable walking distance together with an integrated bike-sharing system [103]. Another study found that potential PT users were more tolerant about PT than regular PT users [56]. The disparity groups in the perception of the types of users regarding the service quality have justified that the PT users were more habitual users than PV users. The main reasons behind the low frequency of PT in regular travel for each group is connected to question (3) that could be answered by the result, where both groups agreed that PT service in Budapest was relatively dissatisfied in terms of cleanliness, noise, space, comfort, and temperature, which concurred with the previous study concerning PV user’s viewpoint about PT service [104]. Moreover, predictably PV users needed a car to transport their kids to school and preferred to use a car (48% and 38%). In contrast, solely 15% of the PT users voted that they needed a car for this purpose, and merely 13%, preferred to use a car.

To answer the last research questions (4 and 5), according to our analysis PV users showed that cost and security attributes need to be the focus of future improvements of PT service providers. This finding was consistent with a previous study that provided sufficient security as one of the main factors in attracting PV users because they felt that the car was more secure than PT [74]. However, for the attribute of cost, previous research discovered that the cost for PT was cheaper than the private car from the perspective of PV users [11]. Thus, new frameworks, such as Mobility as a Service, may solve some cost-related issues by providing optimized mobility packages, including several transportation modes through a single application. Additionally, for people who frequently transfer or re-use the same route, buying a time-based ticket can be more advantageous. Furthermore, increasing the number of security guards at platforms and on trains could provide a reduction in crime.

It was revealed that service hours, frequency, and proximity were well appreciated and serve as the main advantages of the PT service. Thus, the performance of these factors needs to be maintained over time. Nonetheless, the previous study found that PT was not a viable alternative in terms of travel times [105], with slight differences among groups based on the IPA and IPMA results. For both user groups in the IPMA individual space, temperature, and cleanliness were in the low-priority quadrant that supplemented by the open-ended questionnaire results. However, it has to be stated that in general PT system needs to provide good ventilation inside the vehicles and clean stations. These findings are in agreement with previous studies. The results demonstrated that some policies, such as supply expansion and vehicle sanitization, boost people's desire to use PT in the post-COVID-19 era. To prevent crowding and intense vehicle cleaning, increasing service frequency is one of the PT policies that are most widely supported. Likewise, the availability of steering wheel and handlebar covers also considerably boosts people's desire to use shared transport services [71]. Nevertheless, these measures might be a problem for service providers, which is challenging in terms of financing [106]; in other words, it is vital to consider further the economic viability of such measures [71]. The general objective of mobility plan project was that: “the transport of Budapest must improve the competitiveness of the town must also contribute to initiation a sustainable, livable, attractive and healthy urban environment” because, it mostly focused on technical-operational aspects and was not so extensive on the comfort of passengers [78]. Hence, this study will alert PT operators to adopt new policy measures to improve PT services. However, some results and policy implications in this study could be conditioned to the PT system in Budapest. Therefore, the generalization of the results should be taken into account in other contexts because some of the study's conclusions may differ when replicated or used in other locations.

The sample size of the current study was sufficient, and the IPA and IPMA techniques for service quality analysis were used to identify specific service quality improvements. However, additional longitudinal studies would be necessary to better understand how shifting transport modes can affect travel satisfaction and intention over time (i.e., compare attitudes during and after COVID-19). Furthermore, a follow-up study is recommended to incorporate an integrated method to explore the heterogeneity of the different socio-demographic characteristics, for example, age and income. Further possible research might be the development of other DCMs with the IPA and with more disaggregated techniques to obtain accurate results. Further comparison analysis between various nations, for instance, developed and developing countries, is recommended to show the similarities and contrasts in the citizens’ perception of the PT service quality. Moreover, in order to understand the priority of attributes, further research is needed to consider the heterogeneity measurement, and a new survey should be developed using the IPA or an importance-satisfaction model integrated with MCDM techniques.

## Conclusions

This research applies quantitative methods and is supplemented by an open-ended questionnaire as part of the survey analysis for the fulfillment of the research objectives. The satisfaction survey is conducted from two different user viewpoints in Budapest during COVID-19. It should be noted that the outcomes might be influenced by the unusual situation of the pandemic. However, since the occurrence of world-wide pandemic waves cannot be excluded, it might be still relevant more for transport planners and decision-makers.

As the main finding, the perception of the PT users’ satisfaction is higher than the perception of the PV users’ satisfaction regarding the quality of the PT services. On the one hand, the results of the open-ended questions show that both types of users (i.e., PV and PT) choose the main reason for the low frequency of using PT as uncomfortable, noisy, crowded and not satisfactory temperature. Likewise, findings from the OLM demonstrate that the temperature and cleanliness factors are not significant as predictors contributing to the PV and PT users’ general satisfaction. In addition, from the IPMA approach, the factors of cleanliness and temperature are in the ‘low priority’ quadrant. Moreover, there is a general agreement among the groups that the service hour, proximity, and frequency attributes are important. Thus, the PT service providers can keep up the good work on these attributes to contribute to the existing PT users’ satisfaction and attract more PV users to the use of PT. Still, the COVID-19 pandemic and related restrictions may have caused potential bias in the data and changed the user's attitude about PT service quality attributes.

The current study proposes an OLM applying the IPA and the IPMA using the PCA variables and the results of the PLS-SEM to fill the gap of measuring the quality of the PT service. It can be concluded that both IPA and IPMA are performed efficiently. The IPMA is an aggregate means of presenting the service attributes, while the IPA is an appropriate disaggregate approach to display the service attribute priorities. Therefore, the hybrid technique (the IPA and the IPMA) is more integrated than the existing models in the previous literature.

## Data Availability

The datasets used and/or analyzed during the current study are available from the corresponding author on reasonable request.

## Appendix

See Fig. 4.
